# Deciphering the intrinsic dynamics from the beam-induced atomic motions in oxide glasses

**DOI:** 10.1107/S1600577520009753

**Published:** 2020-08-11

**Authors:** Yuriy Chushkin

**Affiliations:** a ESRF – The European Synchrotron, 71 avenue des Martyrs, 38000 Grenoble, France

**Keywords:** XPCS, glasses, beam effect

## Abstract

Probing the microscopic slow structural relaxation in oxide glasses by X-ray photon correlation spectroscopy (XPCS) revealed faster than expected dynamics induced by the X-ray illumination. The fast beam-induced dynamics mask true slow structural relaxation in glasses and challenges application of XPCS to probe the atomic dynamics in oxide glasses. Here an approach that allows estimation of the true relaxation time of the sample in the presence of beam-induced dynamics is presented. The method requires two measurements either with different X-ray beam intensities or at different temperatures. Using numerical simulations it is shown that the slowest estimated true relaxation time is limited by the accuracy of the measured relaxation times of the sample. By analyzing the reported microscopic dynamics in SiO_2_, GeO_2_ and B_2_O_3_ glasses, it is concluded that the beam-induced dynamics show rich behavior depending on the sample.

## Abstract 2

Analysis of the X-ray beam-induced dynamics in oxide glasses probed by X-ray photon correlation spectroscopy is presented. The results offer the possibility to estimate true sample relaxation time by performing two measurements with different X-ray fluxes.

## 1. Introduction   

Upon rapid cooling of a liquid below its melting temperature the liquid may enter into a supercooled liquid metastable state by avoiding crystallization (Doremus, 1994 ▸; Varshneya & Mauro, 2019 ▸). Further cooling increases viscosity and transforms the liquid into a glass at the glass transition temperature *T*_g_. Glasses are amorphous solids and their structural relaxation is too slow to be observed at the laboratory time scale. At *T*_g_ the shear viscosity of a liquid η is 10^12^ Pa s and the structural relaxation time τ is 100 s by convention. According to Angell (1985 ▸), liquids can be classified as ‘strong’ and ‘fragile’. The viscosity of ‘strong’ liquids follows the Arrhenius behavior upon cooling while ‘fragile’ liquids are characterized by non-Arrhenius character. There are several theories that describe the viscosity of supercooled liquids upon cooling (Doremus, 1994 ▸; Avramov, 2005 ▸; Dyre, 2006 ▸; Ojovan, 2008 ▸) but a unified microscopic picture is still missing. During the glass transition no noticeable transformation of disordered structure is observed yet the dynamic structure factor *S*(*q*, *t*) shows a rich behavior that reflects the dramatic slowing down of the molecular motions (Pusey & van Megen, 1987 ▸; van Megen *et al.*, 1992 ▸; Horbach & Kob, 2001 ▸). Therefore study of the microscopic relaxation processes of the glass formation is central to its understanding.

Dynamic structure factors can be measured using scattering experiments. In recent years X-ray photon correlation spectroscopy (XPCS) has been developed as a powerful tool to probe the structural relaxation time at the atomic length scale (Leitner *et al.*, 2009 ▸; Ruta *et al.*, 2012 ▸). In XPCS one measures the temporal intensity autocorrelation function that relates to the intermediate scattering function (dynamic structure factor) (Grübel *et al.*, 2008 ▸). Thus the microscopic nature of the structural relaxation can be investigated. XPCS applied to metallic glasses revealed the dynamical transition at *T*_g_ when the stretched exponential decay in the supercooled liquid state changes to a compressed exponential behavior in the glass (Ruta *et al.*, 2012 ▸). Although the mechanism responsible for the change is not explained, it is a very clear indicator of the glass transition and was observed in numerous studies in colloidal (Kwaśniewski *et al.*, 2014 ▸) and metallic glasses (Ruta *et al.*, 2012 ▸).

Oxide glasses such as SiO_2_, GeO_2_, B_2_O_3_ and their derivatives are an important class of materials from industrial and scientific points of view. Pure silica, germania and boron trioxide are network glasses and their structures are built from the tetrahedron (silica and germania) or linked boroxol groups randomly arranged in a three-dimensional network (Zachariasen, 1932 ▸). The connection of the structural units in the random network is assured by the bridging oxygen atoms. In this respect oxide glasses are fundamentally different from metallic glasses where the atomic interactions are isotropic and hard-sphere like. Moreover, the network glass-formers are ‘strong’ liquids characterized by a high kinetic fragility index (100) while metallic glass-formers have a smaller value (20) (Wang *et al.*, 2004 ▸).

Recent investigations of microscopic dynamics in oxide glasses by XPCS revealed faster than expected relaxation times in a deep glassy state related to the beam-induced effect (Ruta *et al.*, 2017 ▸; Dallari *et al.*, 2019 ▸; Pintori *et al.*, 2019 ▸; Holzweber *et al.*, 2019 ▸). In all of the above cases (Ruta *et al.*, 2017 ▸; Dallari *et al.*, 2019 ▸; Pintori *et al.*, 2019 ▸; Holzweber *et al.*, 2019 ▸) the X-ray probe interacts with the sample and causes artificial structural relaxations that otherwise should not occur in the sample. It was clearly identified that the measured relaxation times scale inversely with the intensity of the X-ray beam (Ruta *et al.*, 2017 ▸; Pintori *et al.*, 2019 ▸; Holzweber *et al.*, 2019 ▸) and that the sample dynamics and beam-induced dynamics are independent processes (Pintori *et al.*, 2019 ▸). Although no visible structural damage was observed during the XPCS measurements, the reported beam-induced dynamics precludes the studies of the true microscopic dynamics in oxide glasses and limits the application of XPCS. The problem will become even more severe with the 100-fold increase in coherent X-ray intensity expected from the synchrotron source upgrade (Raimondi, 2016 ▸) and necessitates further investigation. In this work we address the above issue. Based on the mathematical and numerical analysis we show possible routes to estimate the true sample dynamics from the measured values influenced by the beam effect. The results show that the slowest possible estimated time depends on the accuracy of the measurements.

## 2. Mathematical analysis   

The measured quantity in XPCS is a temporal intensity *I*(*q*, *t*) autocorrelation function, 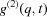 = 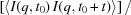 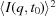, where 〈…〉 denotes the time average over *t*_0_ and *q* is the magnitude of the scattering vector. *g* ^(2)^(*q*, *t*) is related to the intermediate scattering function *f*(*q*, *t*) = *S*(*q*, *t*)/*S*(*q*) via the Siegert relation *g* ^(2)^(*q*, *t*) = 1 + *C*[*f*(*q*, *t*)]^2^, and *C* is a contrast that depends on the experimental setup and sample properties. The intermediate scattering function describes the relaxation process in space and time, 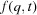 = 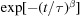 with the characteristic decay time τ, and β defines the shape of the decay. In the supercooled liquid state, β < 1, and in the metallic (Ruta *et al.*, 2012 ▸) and some oxide (Ruta *et al.*, 2017 ▸) glasses, β > 1. τ is the microscopic structural relaxation time of the system and depends on the scattering vector *q* and the temperature *T*. For ‘strong’ glass-formers the temperature dependence of the sample relaxation time τ_s_(*T*) can be described by the Arrhenius behavior 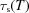 = 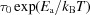, where τ_0_ is a constant, *E*_a_ is an activation energy and *k*_B_ is the Boltzmann constant.

We consider that when beam-induced dynamics occur in a sample, owing to X-ray illumination with flux *F*, the measured relaxation time τ(*F*, *T*) is composed of two contributions: the intrinsic dynamics of the sample τ_s_(*T*) and the beam-induced dynamics τ_ind_(*F*),
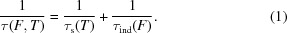
This relation can describe the experimental observations of Pintori *et al.* (2019 ▸) in B_2_O_3_ glass. The beam-induced relaxation time, assumed to be temperature independent, is inversely proportional to the average photon flux (Ruta *et al.*, 2017 ▸; Pintori *et al.*, 2019 ▸; Holzweber *et al.*, 2019 ▸),
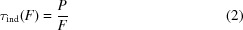
where *P* is a proportionality constant. It depends on a sample’s linear X-ray absorption coefficient μ and sample thickness *L* via 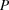 = 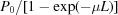 (Pintori *et al.*, 2019 ▸; Holzweber *et al.*, 2019 ▸). *P*_0_ gives a number of absorbed photons that leads to a 1/*e* decay of the intermediate scattering function in a given sample. For the moment we considered *P* to be temperature independent.

Then equation (1) can be rewritten in the following form,
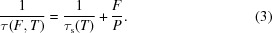
When we have two or more measurements of τ(*F*, *T*) at different *F*, we can fit it with a simple linear model *y* = *b* + *ax*. Then the intercept *b* provides an estimate of the relaxation rate of the sample 1/τ_s_(*T*). This is the basic principle used in the proposed approach but its application is not trivial as we shall see below.

Based on the above principle we can consider two practical cases. The first: at low temperatures, in the deep glassy state, τ_s_(*T*_low_) ≫ τ_ind_(*F*) and we can assume that the measured relaxation time is dominated by the beam-induced effect: τ(*F*, *T*_low_) = τ_ind_(*F*). Conversely, at high temperatures, above *T*_g_, 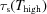 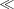 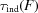 and the measured relaxation time is close to the true sample dynamics: τ(*F*, *T*_high_) = τ_s_(*T*_high_). From the above asymptotic behavior we can envisage measuring the relaxation time at low temperature and use τ_ind_(*F*) = τ(*F*, *T*_low_) for estimating the sample dynamics in the intermediate range near *T*_g_ using 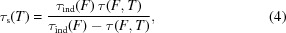
where τ_ind_(*F*) = τ(*F*, *T*_low_). Assuming that τ_ind_(*F*) is temperature independent we can estimate it by measuring the dynamics at low temperature and then use this value to estimate the sample dynamics around *T*_g_. For SiO_2_, GeO_2_ and B_2_O_3_, room temperature can be considered as low enough.

The second case: when the temperature independence of τ_ind_ might not be valid then we can exploit the fact that the beam-induced effect is a linear function of the flux *F*, and by performing measurements using only two different fluxes one can estimate the true sample relaxation time. To accomplish the estimation we must know the ratio *f* = *F*_1_/*F*_2_ (*F*_1_ > *F*_2_, *f* > 1) between two different fluxes used, then it is easy to show that the sample relaxation time can be obtained by
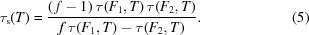
The equations (4) and (5) suggest two ways to estimate the sample relaxation time from measurements. In principle, even in a deep glassy state when the true sample relaxation time is very long to measure during the experiment, the dynamics can still be extracted from the measured beam-induced times. In spite of this interesting possibility, in practice this might be difficult to attain. The accuracy of the measurements imposes the limits on the reliable time estimations.

## 3. Numerical simulations   

**Figure 1:**
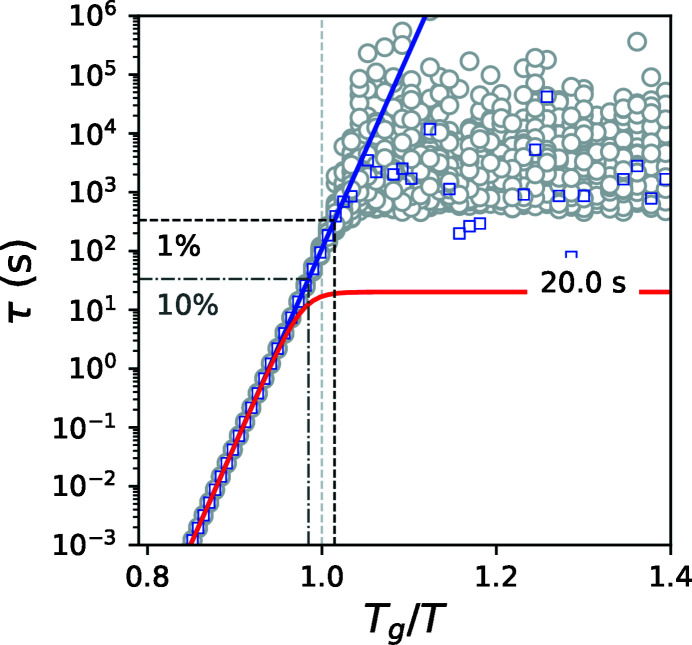
Temperature dependence of the relaxation times. The blue line is τ_s_(*T*), the red line is τ(*F*_1_, *T*) with σ = 1%, gray circles are 100 estimated points of τ_s_(*T*), blue squares are the average of 100 estimated points of τ_s_(*T*), black and gray dash-dot lines are the upper limits for σ = 1% and 10%, respectively.

To study the effect of noise on the estimation of the sample relaxation time we performed numerical simulations. We modeled the experimental noise using a Gaussian distribution with a relative standard deviation σ = 1% of the mean value for τ(*F*, *T*). The temperature dependence of the sample relaxation time τ_s_(*T*) was described by the Arrhenius equation using parameters for *E*_a_ of B_2_O_3_ glass (Ojovan, 2008 ▸) and adjusting τ_0_ to match τ_s_(*T*_g_) = 100 s (*T*_g_ = 580 K) (Ojovan, 2008 ▸). We covered a temperature range across the glass transition region below and above *T*_g_. The ratio *f* was fixed to 10 and τ_ind_(*F*_1_) to 20 s. Using equation (1) we calculated τ(*F*, *T*) adding the noise (red curve shown in Fig. 1 ▸). Applying equation (3) to 100 curves of τ(*F*, *T*) with Gaussian noise σ of 1% we obtained an estimation of τ_s_(*T*) displayed by the gray circles in Fig. 1 ▸. The blue squares are estimated average values. The estimation follows the expected behavior (blue curve) up to an upper limit (black dash-dot line) after which the estimated value flattens.

The flattening results from the behavior of the relative variance 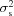 that can be calculated using the following expression,
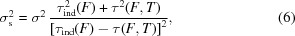
where the same relative standard deviation σ is assumed for τ_ind_(*F*) and τ(*F*, *T*). At low temperatures τ(*F*, *T*) asymptotically approaches τ_ind_ and σ_s_ diverges. Adopting a 3σ criterion that τ_ind_ − τ(*F*, *T*) ≥ 3σ[τ_ind_ + τ(*F*, *T*)] and setting τ_ind_ − τ(*F*, *T*) = 6στ(*F*, *T*) into equation (4) we can estimate the upper limit as 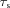 = 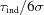. The upper limits for σ = 1% and 10% are drawn in Fig. 1 ▸ for comparison.

**Figure 2:**
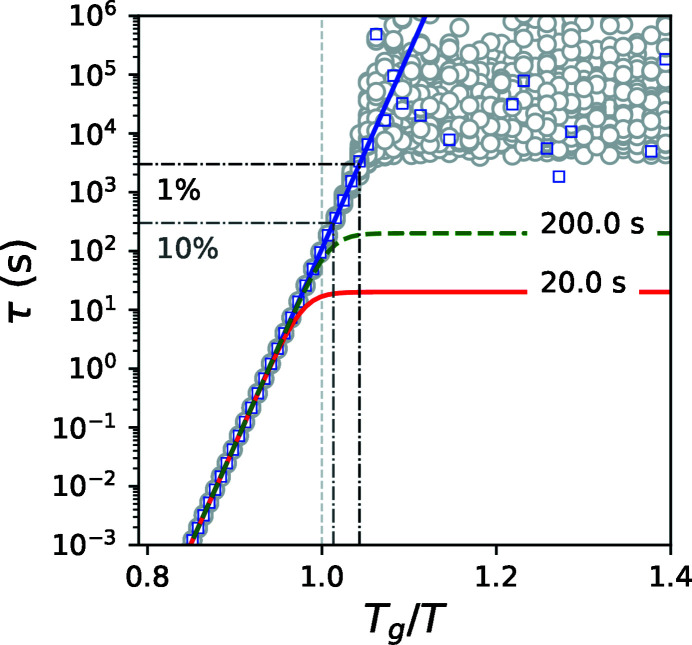
Temperature dependence of the relaxation times. The blue line is τ_*s*_(*T*), the green dashed line is τ(*F*_2_, *T*), the red line is τ(*F*_1_, *T*) both with σ = 1%, grey circles are 100 estimated points of τ_s_(*T*), blue squares are the average of 100 estimated points of τ_s_(*T*), black and gray dash-dot lines are the upper limits for σ = 1% and 10%, respectively.

Now we consider the second case when two measurements with different fluxes are performed at constant temperature. Such a case has an advantage because it can be applied when the beam-induced dynamics might be temperature dependent. The simulated curves (red and dashed green) are shown in Figs. 2 ▸ and 3 ▸. At each temperature we calculated 100 points (gray circles) corresponding to the normally distributed noisy τ(*F*_1_, *T*) and τ(*F*_2_, *T*), both with σ = 1%. At high temperatures gray points are narrowly distributed and coincide with the theoretical value (blue line). At low temperatures we observe a wide distribution of the estimated relaxation time and it deviates from the expected behavior (blue line). The blue squares are average values. At low temperatures the average values deviate from the expected time and saturate at a certain level. The black dash-dot line is the upper limit. The relative variance 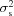 can be calculated using the following expression:
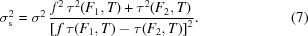
Similar to the previous case, using a 3σ criterion, *f*τ(*F*_1_, *T*) − τ(*F*_2_, *T*) ≥ 3σ[*f*τ(*F*_1_, *T*) + τ(*F*_2_, *T*)] and setting *f*τ(*F*_1_, *T*) − τ(*F*_2_, *T*) = 6σ*f*τ(*F*_1_, *T*) into equation (5), we can estimate the upper limit as 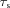 = 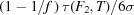.

**Figure 3:**
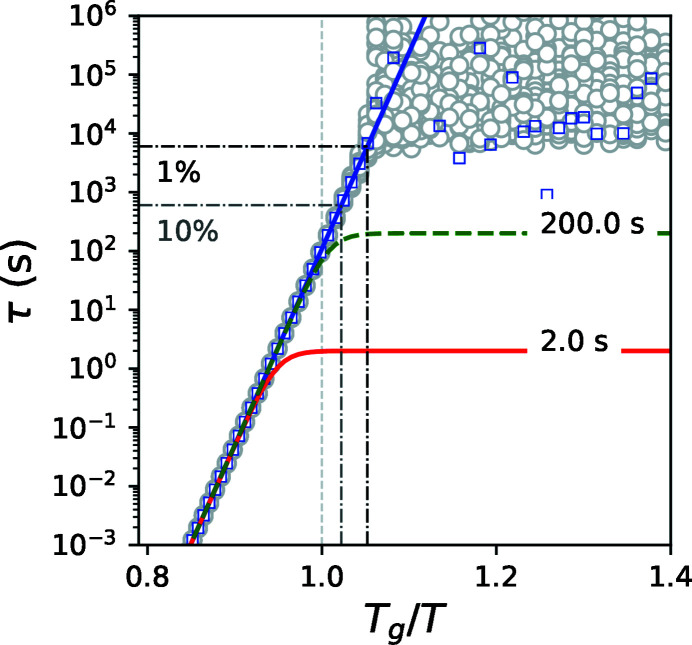
Temperature dependence of the relaxation times with 100 more flux for τ(*F*_1_, *T*) than in Fig. 2 ▸. The blue line is τ_s_(*T*), the green dashed line is τ(*F*_2_, *T*) with σ = 1%, the red line is τ(*F*_1_, *T*) with σ = 0.1%, gray circles are 100 estimated points of τ_s_(*T*), blue squares are the average of 100 estimated points of τ_s_(*T*), black and gray dash-dot lines are the upper limits for σ of τ(*F*_2_, *T*) 1% and 10%, respectively.

These results show that, in practice, the sample relaxation time can be estimated reliably up to a certain time {approximately 16.6[1/(6 × 0.01)] times slower than τ_ind_ or τ(*F*_2_, *T*) when it is measured with an accuracy of 1%}. In the near future, 100 times higher coherent flux is expected from the new diffraction-limited synchrotron sources (Raimondi, 2016 ▸). Higher coherent flux *F* will have two substantial impacts. A positive impact is an increase in the signal-to-noise ratio (SNR) of the correlation function *g* ^(2)^ according to the following equation (Lumma *et al.*, 2000 ▸),
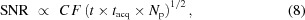
where *N*_p_ is the number of pixels in the detector used and *t*_acq_ is the acquisition time. For simplicity we consider the beam size, sample-to-detector distance, contrast *C* *etc.* to be constant. A negative consequence is that, when the flux *F* increases by 100 times, the beam-induced relaxation time τ decreases by 100 times and hence so does *t*. Nevertheless, the SNR still improves by ten times when keeping the same *t*_acq_. In this condition the relative accuracy of the measured relaxation time will improve by a factor of ten (from 1% to 0.1%) but the absolute values will decrease by a factor of 100. The calculation of this scenario is shown in Fig. 3 ▸. Here we kept τ(*F*_2_, *T*) = 200 s with σ = 1% and set *f* = 100 and τ(*F*_1_, *T*) = 2 s with σ = 0.1%. As one can see, the behavior is qualitatively identical to the previous case but the deviation from the sample relaxation time occurs at a factor of two slower times. Thus, it is possible to estimate the sample time to be 30 times slower than τ(*F*_2_, *T*). This example shows that it is feasible to take advantage of the increase in coherent flux. To obtain the most optimal estimation of the true sample relaxation time within the available measurement time *t*_acq_ one should use the same time *t*_acq_ for both measurements and seek to work with the smallest σ; and *f* should be larger than 15.

## 4. Discussion   

The analysis performed in this study relies on the decoupling of the beam-induced relaxation time from the true sample relaxation time and its strong temperature dependence. It retrieves the sample dynamics without knowing the origin of the beam-induced effect. However, studying both dynamics is important for a better understanding of the glass transition problem and for application of oxide glasses in radiation-high industrial environments. This study addressed the first problem, and the latter one requires further investigation. Recent works (Ruta *et al.*, 2017 ▸; Pintori *et al.*, 2019 ▸) consider radiolysis as the main mechanism of the beam-induced dynamics. An absorbed X-ray photon excites electrons that leads to a transient break up of the atomic bonds (Griscom, 1985 ▸; Ziaja *et al.*, 2015 ▸; Medvedev *et al.*, 2015 ▸) and causes subsequent cooperative atomic rearrangements detected by XPCS. Such beam-induced non-thermal bond breaking produces relaxations similar to thermal viscous structural relaxations. The idea of bond breaking that leads to plastic deformation was used in a model of viscosity of vitreous silica (Mott, 1987 ▸). Several experiments report a connection between radiation-induced bond breaking and viscosity. Indeed, continuous electron irradiation of borosilicate glasses reduced the viscosity and led to fluidization due to non-thermal bond breaking (Ojovan *et al.*, 2009 ▸). Moreover, the electron beam can be exploited for shaping nanoscale vitreous silica (Zheng *et al.*, 2010 ▸). Recently, atomic rearrangements under e-beam illumination have even been imaged in two-dimensional vitreous silica by transmission electron microscopy (Huang *et al.*, 2013 ▸). Plastic deformation (radiation-enhanced viscosity) in amorphous materials has been reported during ion irradiation (Volkert & Polman, 1991 ▸) as well. Understanding the microscopic character of radiation-induced dynamics can be gained by XPCS.

**Figure 4:**
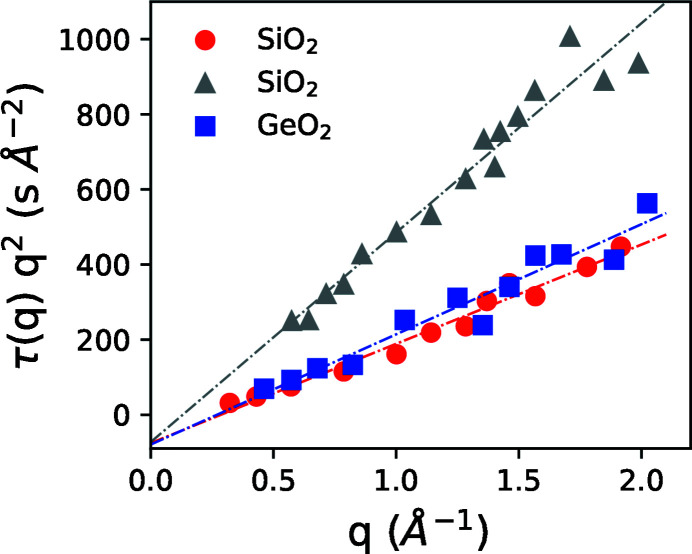
Reduced scattering vector dependent relaxation time as a function of *q* for SiO_2_ and GeO_2_ glasses [data adopted from Ruta *et al.* (2017 ▸)]. Gray triangles are data obtained with a 2.74 times lower flux than the red circles.

Because atomic rearrangements can be sensitive to local structure (Leitner *et al.*, 2009 ▸) it is interesting to look at the length scale dependence of the structural relaxation under X-ray illumination. Previous study provides data of beam-induced relaxation time as a function of the scattering vector (Ruta *et al.*, 2017 ▸). In Fig. 4 ▸ we plot the relaxation time of SiO_2_ and GeO_2_ glasses in reduced units τ(*q*)*q* ^2^. This allows us to compare beam-induced microscopic dynamics with the well understood diffusion process in a colloidal suspension where particles randomly move in a viscous liquid. For a dilute colloidal suspension undergoing Brownian motion the reduced relaxation time is inversely proportional to the diffusion coefficient *D*, τ(*q*)*q* ^2^ = 1/*D* (Pusey, 1991 ▸). When the particle concentration is high and the interaction between them is important, the reduced relaxation time is proportional to the static structure factor *S*(*q*), τ(*q*)*q* ^2^ ∝ *S*(*q*)/*D* (Pusey, 1991 ▸). Such behavior was observed in beam-induced dynamics of alkali borate glasses (Holzweber *et al.*, 2019 ▸). Yet the data for silica and germania presented in Fig. 4 ▸ are markedly different from the above two scenarios. The reduced relaxation time can be better described by a linear behavior τ(*q*)*q* ^2^ = *c* + *kq*, where *c* and *k* are constants. The constant *k* depends on the incident X-ray flux while the parameter *c* is negative and similar for all three curves. The linear behavior and compressed shape of the exponential decay (Ruta *et al.*, 2017 ▸) can be associated with the superdiffusive and cooperative rather than diffusive atomic motions. In addition, the surprisingly negative intercept *c*, that gives a non-physical negative apparent relaxation time, could be a signature of plastic yielding (Volkert & Polman, 1991 ▸), in analogy to a Binghman plastic that contains a negative shear stress term to account for yielding. Yet this conjecture requires further study.

The central premise of the presented analysis is the linear dependence of the beam-induced relaxation time on the inverse of the incident flux that was reported in several experiments on SiO_2_, GeO_2_ and B_2_O_3_ glasses (Ruta *et al.*, 2017 ▸; Pintori *et al.*, 2019 ▸; Holzweber *et al.*, 2019 ▸). The X-ray beam can induce a non-thermal relaxation rate at room temperature that would correspond to the thermal relaxation rate of a glass heated to near its glass transition temperature, thus hundreds of degrees points to a common origin (the role of the bridging oxygen). This suggests a similarity between X-ray induced and thermal transient bond breaking and subsequent bond reformation.

Clearly, in multicomponent oxide glasses the real picture can be more complex. A new study of alkali borate glasses reported a deviation from the single linear behavior in (Rb_2_O)_30_(B_2_O_3_)_70_ glass (Holzweber *et al.*, 2019 ▸). Alkali oxides are network modifiers – their presence in a network glass influences structure, viscosity, ionic conductivity, mechanical properties and glass transition temperature (Varshneya & Mauro, 2019 ▸; Greaves & Ngai, 1995 ▸). In particular, adding alkali oxides in B_2_O_3_ glass transforms trigonal BO_3/2_ into tetragonal 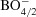 structural units (Varshneya & Mauro, 2019 ▸), leads to microsegregation (Greaves & Ngai, 1995 ▸), formation of ion conduction channels (Greaves, 1985 ▸) and decoupling of ion mobility from glass-forming matrix (Varshneya & Mauro, 2019 ▸). Obviously a simple bond-breaking scenario is not complete and should be elaborated to describe the experimental observations. Moreover, for a new sample, measurements with several different fluxes are required to verify if a beam-induce effect is a linear or non-linear function of incoming flux.

## 5. Conclusions   

The measurements of microscopic dynamics in oxide glasses by XPCS are inevitably affected by the beam-induced structural rearrangements that preclude determination of the true sample structural relaxation time at the atomic length scale. However, when beam-induced atomic motion is a linear function of the X-ray flux, then, by performing two measurements, either at two different temperatures or with two different fluxes, it is possible to estimate the true sample relaxation time in simple glasses up to a certain extent into the glassy state. This extent depends on the accuracy of the measured relaxation times. Therefore, the application of XPCS for the investigation of atomic dynamics in oxide glasses will largely benefit from the increase in precision of measuring the intermediate scattering function, and determination of the relaxation time and the shape parameter. Moreover, the described framework can be useful for developing a better understanding of non-linear beam-induced effects and many intriguing properties of oxide glasses and supercooled liquids around *T*_g_. The observation of diffusive and superdiffusive microscopic behaviors of the beam-induced dynamics and deviation from the linear dependence on the incident flux in various oxide glasses calls for further studies.
